# “And Then Break the Cliché”: Understanding and Addressing HIV Vulnerability Through Development of an HIV Prevention *Telenovela* with Men Who Have Sex with Men and Transwomen in Lima, Peru

**DOI:** 10.1007/s10508-017-1119-x

**Published:** 2018-02-20

**Authors:** Jonathan Garcia, Amaya G. Perez-Brumer, Robinson Cabello, Jesse L. Clark

**Affiliations:** 1 School of Biological and Population Health Sciences, College of Public Health and Human Sciences, Oregon State University, 118C Milam Hall, Corvallis, OR 97331 USA; 2 Department of Sociomedical Sciences, Mailman School of Public Health, Columbia University, New York, NY USA; 3 Asociacion Civil Via Libre, Lima, Peru; 4 Department of Medicine/Division of Infectious Diseases, UCLA Geffen School of Medicine, Los Angeles, CA USA

**Keywords:** HIV prevention, Sexual scripts, Critical consciousness, Men who have sex with men, Transgender women

## Abstract

HIV and other sexually transmitted infections (STIs) continue to affect men who have sex with men (MSM) and transgender women (TW) in Peru at disproportionately high rates. The ineffectiveness of traditional prevention strategies may be due to the disconnect between health promotion messages and community-level understandings of sexual cultures. We conducted 15 workshops with MSM and TW to develop a community-based sexual health intervention. Intervention development consisted of focus groups and scenic improvisation to identify sexual scripts for an HIV prevention *telenovela*, or Spanish soap opera. Workshops were stratified by self-reported socioeconomic status, sexual orientation, and gender identity: (1) low-income MSM (*n* = 9); (2) middle/high-income MSM (*n* = 6); and (3) TW (*n* = 8). Employing a conceptual model based on sexual scripts and critical consciousness theories, this paper reports on three themes identified during the *telenovela*-development process as participants sought to “rescript” social and sexual stereotypes associated with HIV-related vulnerability: (1) management of MSM and TW social identities at the intersection of socioeconomic status, sexuality, and gender performance; (2) social constructions of gender and/or sexual role and perceived and actual HIV/STI risk(s) within sexual partnership interactions; and (3) idealized and actual sexual scripts in the negotiation of safer sex practices between MSM/TW and their partners. These findings are key to reframing existing prevention strategies that fail to effectively engage poorly defined “high-risk populations.” Leveraging community-based expertise, the results provide an alternative to the static transfer of information through expert–patient interactions in didactic sessions commonly used in HIV prevention interventions among MSM and TW.

## Introduction

Controlling the endemic rates of HIV transmission among men who have sex with men (MSM) and transgender women (TW) has been a central but elusive goal for public health systems in Latin America. In Peru, the prevalence of HIV infection among MSM has been estimated between 12 and 22% and among TW as high as 49% (Perez-Brumer et al., 2017; Peruvian Ministry of Health, 2015). Global advances in biomedical prevention have demonstrated great efficacy in reducing HIV transmission risk; however, in Peru, evidence suggests that these strategies are poorly understood and have not curtailed the concentrated burden of disease among key populations (Lee et al., 2015; Ravasi et al., 2016; Sanchez et al., 2007). Existing prevention efforts based on education, outreach, and promotion of HIV counseling and testing among have failed to differentiate between the specific needs of MSM and TW, and have had no observable effect on the local epidemic (Lee et al., 2015; Sanchez et al., 2007). New approaches to prevention that understand and address context-specific meanings of HIV vulnerability and risk among and between MSM and TW are needed to develop appropriate interventions aimed at ameliorating the burden of HIV.

Despite the high prevalence of HIV, perceptions of risk and vulnerability among MSM and TW in Peru are often discordant from their epidemiologic profiles (Clark et al., 2008; Perez-Brumer et al., 2013). In one prior study, 19% of MSM who considered their risk for HIV and sexually transmitted infections (STIs) as “minimal” or “no risk” were in fact HIV-infected, and 17% tested positive for syphilis (Clark et al., 2014). Other research has suggested that there is a population-level discordance between what MSM and TW “know” about HIV risk and prevention and what they “do” in their sexual practices (Beyrer et al., 2013; Garcia et al., 2016a, b). This discrepancy between knowledge and behavior is often complicated by stigmatizing attitudes toward homosexuality and transphobic gender stereotypes that generate barriers to health-seeking and risk reduction (Cáceres, Aggleton, & Galea, 2008). As a result, culturally specific methods that draw on local beliefs, practices and understandings of gender, sexuality, health, and disease are needed to address these discrepancies (Petraglia, Galavotti, Harford, Pappas-DeLuca, & Mooki, 2007).

*Telenovelas* (Spanish soap operas) are central components of Latin America’s cultural imaginary that have been used to motivate public discussions of contemporary social issues like HIV infection, substance use, and intimate partner violence (Blas et al., 2010; Forster, Allem, Mendez, Qazi, & Unger, 2015; Obregon, 2005; Wilkin et al., 2007). *Telenovelas* have also been used as a heuristic approach for participatory research methods through use of role-playing, documentation of cultural idioms, and discussions of the unstated topics underlying social and situational dynamics of vulnerability (Alonso & Koreck, 1999; Forster et al., 2015; McQuiston, Choi-Hevel, & Clawson, 2001). *Telenovelas* are particularly relevant as an HIV prevention tool because they allow sensitive topics, (i.e., sex, sexual orientation, gender identity, sexually transmitted infections) to be discussed in the third person, giving participants the flexibility to observe different scenarios, to act out typical and/or problematic interactions via role-playing, and to ask questions about the topic—all without publicly linking the discussion to their own situation (Paiva, 2000b; Rhodes et al., 2011).

We used the process of creating a *telenovela*-based intervention as a platform to understand and address social contexts and behavioral drivers of HIV and STI risk among MSM/TW in Peru. Our strategy was based on a theoretical framework that promotes cultural reframing and behavior modification as techniques to identify ways to bridge the gaps between perceived and experienced vulnerability and between knowledge and behavior.

### Theoretical Framework

We used theories of sexual scripting and critical consciousness development as a guiding framework. Sexual scripts describe how social norms shape sexual behaviors at the levels of cultural scenarios, interpersonal scenarios, and intrapsychic or intrapersonal scenarios (Simon & Gagnon, 1984). The scripting that takes place at each of these levels shapes how social meanings are assigned to situations and internal sexual desires, thereby developing a shared concept of accepted social and sexual subjectivities. Given that navigation of social situations can vary depending on the cultural scenario in which a behavior occurs, scripting theory allows for analyses of the experiences of MSM and TW separately (in terms of individual decision-making processes) and in combination (in terms of sexual partnership interactions and the formation and maintenance of community behavioral norms). External factors including historical context, location, and culture are all theorized to affect an individual’s sexual development and their scripting of different sexual situations, which allow for participants to reflect on their own histories and integrate these aspects into a culturally relevant intervention. Scripting theory is particularly useful for HIV prevention because it draws attention to changing attitudes toward oral and anal sex, and shifting conceptualizations of homosexuality across time and gender, arguing that sexual acts cannot be understood outside of their specific cultural and historical contexts (Gagnon, 1990). Previous research has highlighted how these scenarios frame the ways individuals learn and relearn to be sexual within specific cultural contexts (Bowleg, Lucas, & Tschann, 2004; Emmers-Sommer & Allen, 2005; Hynie, Lydon, Cote, & Wiener, 1998; Maticka‐Tyndale et al., 2005; Munoz-Laboy, Parker, Perry, & Garcia, 2013). In our analysis, sexual scripting provides a theoretical framework to examine how cultural contexts influence cognitive processes through the intrapsychic scenarios, social relationships, and interpersonal scenarios that influence HIV vulnerability.

Critical consciousness theory has been used as the basis for previous adult learning-based interventions due to its emphasis on critical learning processes like reflection, discussion, and action (Freire, 2000, 2013; McQuiston et al., 2001; Paiva, 2005). Through these multipronged processes, participants “decode” oppressive social norms to imagine alternative individual behaviors and interpersonal relationships (Paiva, 2002, 2005). Contemporary applications of this theory are indebted to Freire’s (2000) reconceptualization of “popular education” as a bidirectional exchange of knowledge, instead of the traditional, unilateral banking of information from expert to patient. Freire drew on local culture to effectively communicate with marginalized populations using *their* symbols, meanings, and language. Using Freire’s model, through the generation of self- and group-level awareness, participants then identify how and why social norms and relationships may affect their behavior. This approach can also be used to understand how relationships of oppression are characterized by the unequal power dynamics that may exist between institutions and individuals, among sexual partners, or within social networks.

Both theories have previously been combined with Latin American notions of empowerment to promote individual behavioral change within sexual scenarios and to create a sense of sexual citizenship (Paiva, 2000a, 2002, 2005). Among Brazilian college students, research using these frameworks has stressed the importance of allowing individuals and groups to analyze sexual scenes and apply social meanings to them in ways that are true to their own understandings and experiences. Paiva’s workshops challenged participants to analyze and decode common sexual situations, and in doing so to generate consciousness and awareness of themselves as actors within the socially available scripts. Notably, these results showed an increase in perceived individual agency over sexual scenes and an increased awareness of external factors that impact the sexual experience.

Other studies conducted in the global South have applied similar conceptual frameworks to better understand structural and systemic disadvantages manifesting as HIV vulnerability in certain populations. Campbell and MacPhail (2002) integrated these theories into a peer education intervention to reduce high rates of HIV among adolescents and young adults in a South African community. They argued that arriving at “critical consciousness” required an important change from *intransitive thought* to *critical transitivity*—through which individuals could critically think about their condition and position within the specific social framework of their culture and location. Despite the external factors limiting the observed effect of Campbell and MacPhail’s study, their research suggested that collaborative sexual education, as opposed to didactic methods, would be more successful in raising awareness of HIV/STI vulnerability while also generating a stronger sense of individual and community efficacy.

While critical consciousness theory has previously been used to reframe the gendered power dynamics that make young heterosexual women more vulnerable to HIV (Campbell & MacPhail, 2002; Paiva, 2005), this framework has not been used to explore how local contexts of gender, sexuality, and HIV-related vulnerability are understood and addressed by MSM and TW in Lima, Peru. Existing prevention strategies in Peru are often based on a static transfer of information through expert–patient interactions in structured, didactic sessions, directed toward poorly defined “high-risk populations.” This approach does not account for experiential knowledge from communities most impacted by HIV and, as such, is limited in its ability to effectively engage socially marginalized groups. Addressing this limitation, this paper reports on themes identified during the *telenovela*-development process as participants sought to reconfigure, or “rescript,” social and sexual stereotypes associated with HIV-related vulnerability among MSM and TW. Using a community-based approach that leverages existing community expertise for the holistic development and dissemination of prevention knowledge, we present narratives from these socially marginalized communities most burdened by HIV and AIDS to better understand specific social, cultural, and interpersonal contexts of HIV vulnerability and inform sustainable public health strategies in urban Lima.

## Method

From June to July 2012, we conducted three 5-session sets of weekly workshops (15 sessions total) to collaborate with socially and economically diverse groups of MSM and TW in Lima, Peru in the development of an HIV prevention *telenovela*.

Lima is the second largest city in South America, with close to 10 million inhabitants (INEI Peru, 2016). The urban landscape is divided into 43 metropolitan districts; however, the political geography within the city is characterized by spatial segregation in which the majority of wealth, healthcare, and other public amenities are concentrated within less than 10 districts (Peters & Skop, 2007). While most adults receive at least a secondary education, quality of education varies greatly based on district of residence (Hall & Peters, 2003). Differential access to social services has also been associated with health disparities in the LGBT community. High levels of discrimination, poor specialized knowledge among medical providers, and structural barriers stemming from enforced binary cisgender identity in legal documentation have been identified as determinants of LGBT health disparities in Peru (Maiorana et al., 2016). Most Peruvians are adherent to Christian religions (81% Roman Catholic; 15% Evangelical), which is reflected in social tensions and conservative policy agendas that constrain health and services for LGBT persons (Caceres, Cueto, & Palomino, 2008). Additionally, recent literature has documented the unique emergence of role-based sexual identities among gay men in Lima (Clark et al., 2013) and intersectional identity statuses (e.g., religious affiliation, positionality on a gender spectrum, age, race) among transgender women (Salazar, 2016). To better reflect Lima’s distinct social, political and economic urban landscape we included socially and economically diverse groups of MSM and TW in our workshops.

### Participants

We used purposive sampling (Palys, 2008) to form three subgroups of MSM and TW along the axes of self-reported socioeconomic status, sexual orientation, and gender identity. To be eligible, potential participants needed to report: that they were assigned a male sex at birth, self-identified as male-to-female TW or elsewhere on the trans-feminine continuum (e.g., “trans”, “transgender”, “travesti”), had interest in participating in acting-based workshops, and that they were 18 years or older. Participation was not restricted by reported HIV serostatus. Participants who anticipated not being able to attend the workshop sessions were unwilling to participate in group discussions or acting exercises, or were unable to provide informed consent were excluded from participation.

We conducted venue-based recruitment. Participants were recruited by printed and electronic flyers distributed to local gay and trans community centers in Lima for posting in the center and dissemination to their Internet sites and mailing lists. Upon contact by the potential participant, peer health promoters—who were Peruvian, identified as gender and/or sexual minorities—provided information about the study objectives and requirements and confirmed continued interest in participating. Participants were asked to contact the study office by telephone or by email. The study had a local telephone number and a designated email account to provide information about the study and communicate with participants in the screening procedures. Study staff determined eligibility using an IRB-approved screening and recruitment script. Screening took place by telephone, or in person for potential participants who preferred to visit the study office. Workshops were stratified according to self-reported socioeconomic status, sexual orientation, and gender identity: (1) low-income MSM (*n* = 9); (2) middle/high-income MSM (*n* = 6); and (3) TW (*n* = 8).

### Procedure

Workshops were designed as formative research for a future *telenovela* to address issues of stigma, discrimination, empowerment, and HIV/STI prevention among MSM/TW. The workshops were facilitated by a multi-disciplinary team which brought together expertise in social and behavioral research and Theatre of the Oppressed (Boal & McBride, 2008). Study events, including improvisations and focus group discussions, were led by a team of 2–3 research staff members. Bilingual facilitators had extensive experience in qualitative research with the LGBT community in Lima. Each group’s first meeting focused on development of three primary *telenovela* characters. Subsequent weekly workshops used each group’s three characters as the principal actors in role-play scenarios to address: HIV/STI testing; partner notification and serostatus disclosure; condom negotiation within diverse partnership contexts; and experiences of homophobia/transphobia, stigma, and discrimination. Additional secondary characters were introduced by the facilitators as needed, based on specific topics discussed. Each workshop session followed a similar process-based format: (1) presentation of the theme and discussion of common issues in a traditional focus group discussion (FG 1; 20–30 min); (2) role-playing/character-based enactments of typical behavior or interactions (RP 1; 20 min); (3) decoding discussion of role-play scenarios to identify problems with typical behavior and brainstorm alternatives (FG 2; 20–30 min); and (4) additional role-playing to test alternative scripts incorporating the group’s proposed solutions (RP 2; 15 min). The goal of the focus groups was to initiate discussion of the workshop’s main topic(s), to establish the parameters of the session, and to identify key problems of each topic as related to HIV and STI prevention. Focus group probes aimed to promote participant–participant interactions and to elicit a reflexive understanding of the social discourses shaping individual and shared experiences. A facilitator with theater experience guided role-playing exercises and discussions to provide an alternate format for articulating the key problems of each session’s selected topic. The use of each group’s collectively developed characters as the basis for improvised representations allowed participants to enact typical risk-associated interactions in a socially acceptable format. Through this approach based in critical consciousness theory, participants were guided to decode dominant understandings of gender and sexuality by practicing skills for altering traditional sexual interactions and literally produced new scripts for social interactions. Written informed consent was collected from each participant prior to the start of the first session. Participants received 20 *Nuevos Soles* (~US$ 7) as compensation for attending each session and a bonus of 100 *Nuevos Soles* (~US$ 35) if they attended all of the sessions. Institutional Review Boards at Asociación Civil Via Libre in Lima, Peru, Oregon State University, and UCLA approved the study.

### Theoretically-Informed Analytic Approach

Integrated use of role-playing and focus group discussions resulted in a process of iteration, reiteration, and transformation of the social and cultural representations of gender, sexuality, and HIV/STI risk among Peruvian MSM and TW. Our analysis was guided by immersion–crystallization (Borkan, 1999; Ellingson, 2009), involving both inductive and deductive approaches to identify salient themes related to our theoretical framework, other themes that emerged organically from the data, and the relationships between themes. To organize our findings, we applied conceptual frameworks of sexual scripting and critical consciousness to identify consistencies and discordances between selected themes. Three authors collaboratively constructed the codebook by first creating an initial “start-list” of a priori codes based on the focus group guides and role-playing probes; then, all coders coded 5 transcripts (10% of total), generating a list of “open codes”; coders met to generate consensus about the list of emergent codes to ensure that it was parsimonious, and to ensure that code definitions were accurate, capturing theoretically similar narrative excerpts. The coders subdivided and coded the dataset, meeting weekly to discuss substantive memos and to make adjustments to the codebook (e.g., eliminate redundancies in codes) using Dedoose version 5.0.11 (2014, www.dedoose.com). Analysis of 50 transcripts of pre- and post-improvisation focus groups (FG 1 and FG 2) yielded 37 codes related to how stereotypes were used across social spaces and how cultural norms and beliefs generated situational and contextual HIV vulnerability. During this process, we organized codes and sub-codes into overarching themes that emerged from triangulating qualitative data generated in each workshop, including traditional focus group discussions (FG1), role-playing improvisation of scenes derived from the workshop’s topic (RP1), and concluding group discussions (FG2) and additional improvisations to recognize and resolve discordances between the initial focus group discussions and subsequent role-playing exercises (RP2). In presenting excerpts from transcripts, we identify the socioeconomic, sexual, and gender identity of the group as well as whether it occurred during pre- (FG1) or post- (FG2) role-playing exercises.

## Results

We present our results in three thematic groupings: (1) managing sexual and gender identities across social contexts, geographic venues, and partner interactions; (2) decoding how HIV/STI risk is perceived in relation to sexual role and gender identity; and (3) the impact of these factors on sexual and gendered scripts for negotiating safer sex practices. Below, we discuss each of these themes in greater detail and explore how different qualitative methods elicited distinct understandings of social space, cultural norms, and beliefs related to HIV/STI vulnerabilities.

### Managing Social Identities at the Intersection of Socioeconomic Status, Sexuality, and Gender Performance

Pre-improvisation (FG1) narratives demonstrated how both MSM and TW adopted and reinforced self-stigmatizing attitudes. During these discussions, MSM and TW identified “strategies” they used to “manage” their publicly presented identities and avoid homophobic and/or transphobic discrimination while negotiating the shifting social, geographic, and temporal contexts of their daily lives. Collapsing together distinctions between gender, sexuality, and socioeconomic status, both subgroups of MSM (i.e., low SES and middle/high SES MSM) associated being “careless” with being “feminine,” having “little education” and with using “the gay community’s slang.”

P1: Sometimes you can’t hold it, the truth about your sexual identity all escapes, but when you go to different places, depending on the space, you manage your way around the situation.

P2: Of course, you behave according to the space, you behave like others around you.

P3: Of course, if you’re straight-acting, you can pass amazingly, but if you’re flamboyant [*loca*], careless and feminine, then you can’t.

P4: Maybe this will sound discriminatory: I’ve always thought that it’s an advantage to accept ourselves, but to avoid appearing feminine in public. (Middle/High Income MSM, FG1)

I think discrimination happens a lot if you have little education. If I use language correctly, I’ll be accepted, it shows I may have money. And I need to watch my use of slang (*jerga*), which the gay community uses a lot, because it will give you away. I think the community’s slang will identify you; they’ll reveal you as a crazy queen (*loquita deschavada*). (Low Income MSM, FG1)

Similar to the association of socioeconomic class with stereotypical flamboyance among MSM, TW expressed internalized transphobia in linking a failure to “pass” with a “lack of culture and manners.” Though conscious of the inappropriate challenges TW face in maintaining heteronormative standards of behavior, TW participants stated that the solution was for TW to “work harder” to fit in by better locating themselves within masculine/feminine gender binaries and by adhering to socially acceptable norms of femininity.

We encounter discrimination and transphobia on a daily basis, but I think it has to do with a lack of culture and manners on our part, too. We [TW] have to understand that, living with a taboo, we have to work harder to be valued as humans more than anything. But we also play a part in situations when we’re disrespected, we seek negative attention, causing scandals. We should act more like ladies, so that people will respect us. If we cultivate respect, people will value us.” (TW, FG1)

As evident in these narrative excerpts from FG1 discussions, managing public presentations of gender and sexuality was linked to socioeconomic status on both a cultural and a structural level. In contrast, in post-improvisation discussions (FG2) both MSM and TW decoded the self-stigmatizing words that they used (e.g., “*loca deschavada*” to denote one who has revealed their identity in an unhinged way, and “*maricona ahombrada*” to denote a transgender woman who has failed to be effectively feminine), as exemplified by the following excerpt.

It happens like this. We are a group of friends, half of us are trans and the other are identified as men, some of the *deschavadas* wear a woman’s blouse and a man’s pants. In this group, there’s discrimination among us. For example, the trans woman who has her whole body done, with breasts and hips, will tell the “manly faggot” (*maricona ahombrada*) that they are not good enough to be *trans*. She calls him ugly, makes that person feel worse so she’ll feel better. That’s how she’ll manage the value of her image. A lot of us do it. And in the group, if someone has money, their femininity will be tolerated better. In some groups of friends, it’s all about money, if they are sex workers competing to capture clients. If I’m in a group with all masculine guys, then we pick on anyone with any femininity. (Middle/High Income MSM, FG2)

MSM who identified as “low-income” also noted the reverse process where higher SES resulted in greater social acceptability regardless of gender or sexual identity: “if you are white, good-looking and have money, you fit into any social group. You are less likely to be discriminated against in groups with other gays” (low-income MSM, FG2).

After reflecting on the socioeconomic and cultural origins of their vulnerability in RP1/FG2, TW explained:

Discrimination against the trans population comes from everywhere in society – from your family at home, if you are a sex worker in the streets, always fighting with the police and security (*serenazgo*). Just for dressing as women, they say we are scandalous, don’t stop their car when we’re crossing the street and yell. At the medical post, the nurses laugh with doctors about trans clients; doctors don’t call us by our social name, how we feel and like to be called. For example, if my name were Miguel or Juan Carlos, and even if they see me as a woman, they’ll call me a man’s name at my medical appointments. It’s not just that we are scandalous. (TW, FG2)

As highlighted above, participants recognized that it was not being “scandalous” that made TW vulnerable, but instead underlying structural discrimination that systematically limited their access to safer ways to make money other than sex work, made them targets of police brutality, limited educational opportunities, and made seeking medical care more difficult. All participants became critically aware of the need to dissolve the cultural association of gender transgressive behavior with a “lack of education” or as “badly cultured.” These shifts in self-understanding observed from FG1 to FG2 suggest that, through role-playing, participants recognized how their insults undercut their gender legitimacy (i.e., what it means to be feminine, or the correct way to reveal oneself, *deschavar*) of other MSM and TW. Using an improvisational role-playing format, participants began to develop a critical consciousness of the socioeconomic origins of their vulnerability and to untangle internalized stigma from the social and structural contexts of risk.

### The Impact of Gender and/or Sexual Role Constructions on Perceived and Actual HIV/STI Risk(s) Within Sexual Partnership Interactions

The homophobic and transphobic language that participants used in pre-improvisation groups to police norms of gender and sexuality also mapped onto their perceptions of partner-specific HIV/STI risk. Stigma and disease were typically ascribed to the members of the MSM and TW communities who were seen to violate acceptable boundaries of behavior. TW reported, “They are at risk if they are promiscuous, careless, and crazy women, the ones that are so extreme and always hungry for sex.” Among low-income MSM, men who played the *pasivo* (receptive) or “feminine” role during anal intercourse were considered most at risk since, “We know that if you are *pasivo*, you have a higher probability of getting infected.” In contrast, for middle/high-income MSM, the greatest risk for HIV transmission was found in boundary-crossing contradictions between a person’s inner sexual desire and their publicly presented sexual identity:

The ones most at-risk are men who have wives and girlfriends, and they have sex with men on the side. They are unfaithful, say they don’t want to be contagious for their wives, but continue having sex with men. They are the “frustrated faggots” (*maricones frustrados*). They only get married to hide their homosexuality, and they end up getting infected using the saying “my ass is for men to use and my penis is for women.” (Middle/High Income MSM, FG1)

During subsequent improvisations, participants identified larger societal shifts in messages about HIV and mortality and began to “decode” their individual perceptions of HIV risk. Participants recognized and challenged common representations of non-gay-identified *activo* (insertive partner during anal sex) MSM as virile and STI-free and their gay-identified or transgender partners as promiscuous carriers of disease.

P1: I think it’s true that generally, for example, when an *activo* finds out he was infected, he thinks that the *pasivo* partner infected him.

P2: Totally *machista*.

P1: And the *pasivo* is also *machista* because he doesn’t think that it was the *activo*’s fault, but blames another *pasivo* partner that the *activo* had before. So, there is *machismo* from both *activos* and *pasivos* themselves.

P2: True, generally all *activos* think *pasivos* have infected them. (Middle/High Income MSM, FG2)

To rewrite these social norms, participants identified a need to move away from messages that have been “implanted like computer chips” about the inevitability of HIV infection for MSM and TW. By rejecting the fatalistic link between homosexuality and AIDS, participants articulated the importance of recognizing HIV as a common, but manageable, risk for themselves and their community.

We are reluctant to take an HIV test. We have to take into account what has been implanted like computer chips, since we were little kids: That only homosexuals get HIV, only prostitutes get it, and that it’s the disease you will die of. Now we need to start changing that computer chip, understand that anyone can get HIV—that we are all vulnerable. It’s simply a chronic disease that we can get. (TW, FG2).

As participants identified and sought to change the preconception that only *pasivo* MSM and TW are vulnerable to HIV, they accepted the importance of sexual role in defining the mechanical risks of HIV transmission while also stripping away the accumulated social residue that aligns gender and sexual identity with HIV status. During one post-improvisation discussion about whether to define a specific character’s previously ambiguous sexual role, one lower-SES MSM participant stated:

I think we need to know the character’s sexual role; it’s not a cliché. It’s simply a way for us to understand the person, what kinds of sex he has, and his psychological profile. We can’t just pretend that sexual roles don’t exist or that they don’t matter in managing our daily sex lives. In our daily stories, we think about risk in terms of sexual role; if you’re *pasivo,* you’re more at risk for infection. And we can also play with the role of the characters to challenge our prejudice that only *pasivos* are at risk, and then break the cliché. Perhaps the character can be *moderno* (versatile) and got infected because he had low self-esteem, identity problems, maybe he was a homophobic gay. You understand? He rejected himself, but was still exposing himself in bed. The gay mindset can be neurotic and complex and rich. (Low Income MSM, FG2)

Critically evaluating how standardized public health messages (“computer chips”) used gender and sexuality as proxies for transmission risk to categorize all receptive partners as “at risk” encouraged participants to dissect social constructions of deviant sexuality from biological processes of infectious disease transmission. At the same time, they began to recognize how the internalization of these stigmas could lead to a feeling of “AIDS fatalism” (Diaz, 1998) and further increase their risk for HIV acquisition. As a result of this critical reinterpretation of social constructions of gender, sexual identity, sexual role, and HIV transmission, participants started to develop the discursive language and collective understanding needed to address HIV as a discrete, manageable health risk rather than a poorly defined and inevitable threat.

### Idealized and Actual Sexual Scripts for the Negotiation of Safer Sex Practices Between MSM and TW and Their Partners

The third emergent theme builds on the question of how standards of gender, sexuality, and sexual partnership are maintained, managed, and decoded in scripting sexual practices. During FG1 discussions, participants described adhering to recommendations for routine condom use, while also allowing for differences in scripted sexual practices according to the type of partnership (i.e., steady boyfriend, one-night-stand, commercial sex client). For partners that they trusted and loved, their main HIV prevention strategy consisted of “going together to take a test [for HIV/STIs] before engaging in sex without condoms” (low-income MSM, FG1). Participants who engaged in sex work described the idealized sexual script of “always using condoms with clients” but not consistently with their boyfriends. As one TW explained, “I prefer taking care of myself [using a condom] with people on the street, not with my partner…That’s why if 1 day I get infected, I’m not going to blame my clients.” (TW, FG1)

Both social and geographic factors were reported to influence negotiations over condom use. Among middle/high-income MSM, one of the main drivers was the venue for the contact, with nonverbal interactions and the social conventions of public sex guiding negotiations over sexual practices.

P1: They began their negotiation, in their own language, about how to protect themselves, accepting and rejecting different aspects of sex. They showed us how to behave in a sauna with someone you’re attracted to.

P2: Most of the time you don’t actually talk about negotiation, it just happens, without words. Everyone is in one place, the space is full of steam, and men with towels. They touch each other, and begin having oral sex. They change positions, and in that moment, there is no exchange of words, not even a hello. They get up from there, nod, and proceed to a small room that are specifically for hooking up. When they are finished, they don’t say goodbye.

P1: And that first contact is flirtation, having oral and anal sex; they don’t worry about condoms; they just go on with sex.

P2: I’ve seen that they use nothing, they lower their towels and just go on, with the next expected motion.

P1: They gaze at each other in the eyes first, practically like this [shows others how], and the other accepts or rejects sex—because sometimes you’re just there to relax.

(Middle/High Income, MSM, FG1)

The majority of low-income MSM and TW, among whom commercial sex was commonly reported, expressed the belief that most people in their communities would have condomless sex if offered more money—a perception that contradicted their own stated practices of always using condoms with clients. In these situations, practical issues of money, time, and space would sometimes lead participants to bargain away protections against HIV and interpersonal violence:

So, they go to the client, and there is a negotiation. When the client comes, and wants to have fast sex, in the streets or somewhere public—we don’t just have sex in hotels—if it’s in the street, I can’t really think about condoms, or try to convince him. He says 25 *soles*, I do it without a condom for that, and we part ways. When we have a hotel room, then there is room for negotiation, and things are done differently; I feel I can ask for a condom. (TW, FG1)

During RP1 improvisations and FG2 discussions, participants discussed how the nonverbal cues they use to initiate sex could also be used to improve safer sex negotiations. Referring to sex in the sauna, MSM identified “unspoken” negotiations using “touch” and “gazing,” which often ended in condomless sex. However, participants also agreed that nonverbal cues could be central to initiating safer sex (e.g., carrying a condom, showing it to a partner prior to sex, or simply putting it on without discussion), especially in silent spaces where verbal interaction is not a part of the script. Participants also reflected on how these negotiated sexual situations could offer other opportunities to reduce vulnerability, for example by “using sexual desire to manipulate men into using condoms” (low-income MSM, FG2) in the dark room.

P1: You slowly tempt the other person, and they give in a little.

P2: Yes, you have to consider that both guys are sexually excited; that’s what they’re there for, to look for sex.

P1: They are there to have sex without a condom or whatever comes their way.

P2: If you’re in a dark room, you don’t know who you are with, so it’s all about touch, it’s not even visual.

P1: So, if you make them really horny, you can make them use a condom; just slip it on when they’re really hard. (Low Income MSM, FG2)

Through this strategy of making partners feel “really horny” or more “sexually excited” so they would not refuse condoms, participants reversed dominant sexual scripts linking uncontrollable *activo* male sexuality with condomless sex. Recognizing the greater likelihood of HIV transmission through receptive anal intercourse while addressing the social norms constraining condom use by receptive partners, middle-income-identified MSM identified “tactics that *pasivos* could use to make *activos* use condoms.” (Middle/high-income MSM, FG2)

P1: For example, I can start to caress him like this, softly kiss him; I start to warm him up, and the *activo*, being hot, can’t go back to having control because he wants to go forward towards sex. That’s when they accept wearing a condom; because they’re hot, and they don’t want to go backwards. So, you take advantage and you put a condom on him.

P2: That’s how you manipulate them (laughs). (Middle/High Income MSM FG2)

P1: Tell me, what are your tricks?

P2: When you arrive in the room, have your client lie down, and start giving him a blow job, without a condom. Then you put the condom in your mouth, and clients can’t tell you’ve slipped a condom on them until the end. (TW, FG2)

Sexual desire and eroticism, across groups, emerged as tactics for incorporating safer sex practices (e.g., condoms) within established norms of sexuality and sexual roles. Participants discussed ways that those who were often “blamed” as “irresponsible risk takers” could reframe their understanding of responsibility and reverse power relations by redirecting the “uncontrollable” sexual desire of *activo* partners as tools for their own protection.

## Discussion

Our analysis addresses two foundational challenges for sexual health promotion and HIV prevention among MSM and TW in Peru. First, it presents a novel multipronged qualitative methodology that epistemologically addresses the disconnection between perceived and actual HIV vulnerability. Second, it lays out key insights concerning how cultural dynamics that characterize oppressive relationships can be decoded and rescripted through improvisational role-playing. Social and sexual relationships were shaped by cultural dynamics along the interrelated axes of social class (e.g., being perceived as educated), gender performance, and sexual role. The results suggest that our telenovela-development intervention can be conceptualized as a form of sexual scene practice where participants begin to witness, discuss, and enact multiple social scenes, to develop their own ideas of identity and sexuality, and to begin to analyze and understand the factors that shape their sexual behavior in partnership contexts. Our telenovela-based intervention is the first in Peru to build on adult learning and social science theories to emphasize critical learning processes (i.e., reflection, discussion, and action) to combat experiences of internalized stigma. The expertise of our workshops’ MSM and transgender women participants was central to development of the intervention and, as such, emergent themes incorporate linguistically and culturally appropriate strategies for these key populations in Peru’s HIV epidemic.

Building on research in Latin America and among Latino populations in the U.S. (Clark et al., 2013; Rhodes et al., 2011) our data show how social class, gender performance, and sexual roles together shape interpersonal and cultural dynamics within MSM and TW sexual partnerships. Role-based sexual and gender identities were described as structuring the mechanics of sexual practices, the interpersonal dynamics of partnership interactions (including control over use of condoms and other prevention tools), and partner-specific perceptions of HIV/STI acquisition and transmission risk. Performed indicators of social class, which involved acting “educated” and “well-cultured,” often intersected with conformity to social boundaries of gender, and perceived HIV/STI risk. This observation suggests that effective prevention interventions need to understand and address the diversity of gendered sexual identities circulating among Peruvian MSM and TW, the interplay of these identities with SES in sexual partnerships, and the resulting impact on uptake and use of different HIV/STI prevention modalities.

A growing literature describes the social and cultural factors that affect perceived risk and that generate fatalism (i.e., perceived inevitability of HIV infection) among Latin American and Latino MSM and TW (Diaz, 1998; Mauk, Perry, & Munoz-Laboy, 2013; Severson et al., 2013). Research indicates that homophobia, transphobia, gender discrimination, and class discrimination collectively generate a perception that HIV infection is inevitable, and disincentivizes MSM and TW from engaging with HIV prevention. Studies on sexuality- and gender-related stigma in Latin America have focused primarily on fixed notions of sexual roles (*activo* and *pasivo*) and static masculine ideologies (*machismo* and *marianismo*), but more recent studies have addressed how changing sexual roles and gender fluidity affect engagement with health services and HIV prevention (Clark et al., 2013; Klein, 1999; Padilla, 2007). Our analysis builds on this work to deconstruct sexual and gendered stereotypes and suggest ways in which Peruvian MSM and TW can co-create a critical consciousness approach to revise sexual scripts and “break the cliché” for HIV prevention.

Notably, our novel use of guided role-playing intended to generate critical consciousness about stereotypes related to gender and sexuality highlighted the disconnect between “top-down” health education approaches and how they were understood, embodied, and enacted within MSM and transgender women’s communities. The results presented here link to previous literature underscoring the challenges that sexual stereotypes and dissonance in risk perceptions present for safer sex promotion among gender and sexual minorities (Garcia, Muñoz-Laboy, Parker, & Wilson, 2014; Perez-Brumer et al., 2017; Rhodes et al., 2011). Within the Latin American context, a critical consciousness approach seeks to oppose the notion of “banking education” which often strips communities of agency by imposing strategies that public health experts deem to be more effective. In the words of our participants, messages “implanted like computer chips” reproduce social stereotypes and obscure the social meaning of “high-risk groups.” Through the multipronged process of telenovela development (e.g., listening, discussing, acting, and reflecting), participants began to identify and decode interpersonal scripts that perpetuated HIV stigma in their social networks and affected how they perceived their own and others’ risks and vulnerabilities to HIV.

These alternative scripts emerged in what is described as a point of critical transitivity (Campbell & MacPhail, 2002)—i.e., understanding the socially constructed links between sexual roles, gender performance and socioeconomic vulnerability. Our results highlight the ways in which participants grappled with traditional social “clichés” inherent in stereotypes like uncontrollable gay or transgender sexuality by deconstructing the stereotype of the “*loca pasiva*” (“crazy bottom”) while identifying the internalized stigma and conflicted identity of new archetypes of risk like the closeted bisexual *activo*. Our data also highlighted how participants strategically rescripted stereotypes about sexual desire (e.g., uncontrollable sexual desire commonly considered a masculine trait, wiliness, and manipulation of this hot-tempered sexuality is often considered feminine) to entice their partners into condom use. These sexual scripts were dependent on geographic space (e.g., sauna versus street-based sex). Unique to this analysis are the rich social, cultural, and historical roots of sexuality, gender identity, and sexual behaviors described by participants that suggest the potential for reshaping behaviors and attitudes toward sex, including safer sex behaviors. Jointly, these results support the call for *prevention literacy* based in theoretical frameworks developed in the global South, to not only partner with communities most impacted by HIV but recognize and affirm community-based expertise to shape pedagogical approaches to HIV prevention and health promotion (Campbell & MacPhail, 2002; Paiva, 2005; Parker et al., 2016).

There are important limitations to consider when interpreting our results. Data are drawn from a formative qualitative study and may not be generalizable. The aim of the study was to generate characters and storylines for a *telenovela*-prevention intervention drawing from different subgroups of low-income MSM, middle-/high-income MSM, and transgender women. Due to our stratified study design, we were unable to assess potential intersections between these groups to specifically analyze how inter-community dynamics or sexual partnership interactions across different subgroups may influence HIV prevention behavior. Future qualitative work is needed to detail the complex networks within and across MSM and TW in Peru and how this social landscape can be leveraged to further support HIV prevention efforts. Despite these limitations, our results provide an important first step in informing the development of novel health promotion strategies in collaboration with MSM and TW that incorporate linguistically and culturally appropriate strategies for these key populations.

Ultimately, the dissonances between knowledge and behavior, and theory and practice, parallel a global paradox in which HIV biomedical prevention has rapidly evolved while community-based efforts to make these methods acceptable, accessible, and sustainable for most at-risk groups remain insufficient (Parker et al., 2016). To better support biomedical advances, additional research is needed into the cultural norms and beliefs that influence gendered sexual identities, sexual partnership interactions, and the uptake of specific HIV prevention methodologies (Garcia et al., 2016a, b). The participatory methods outlined in this article are important for defining future research to explore how HIV risk and prevention are understood, recognized, managed in the shifting contexts of daily life among MSM and TW in Latin America. The resulting process through which MSM and TW participants began to replicate, decode, and transform the scripts guiding HIV prevention will be essential to future critical consciousness interventions with these communities.
